# Trends in Illicit Cannabis Potency based on the Analysis of Law Enforcement Seizures in the Southern Area of Rome

**DOI:** 10.3390/toxics11080648

**Published:** 2023-07-26

**Authors:** Francesca Vernich, Lucrezia Stefani, Denise Fiorelli, Federico Mineo, Margherita Pallocci, Michele Treglia, Luigi Tonino Marsella, Roberta Tittarelli

**Affiliations:** 1 Laboratory of Forensic Toxicology, Section of Legal and Forensic Medicine, Social Security and Forensic Toxicology, Department of Biomedicine and Prevention, Faculty of Medicine and Surgery, University of Rome “Tor Vergata”, Via Montpellier 1, 00133 Rome, Italy; 2 Department of Biomedicine and Prevention, University of Rome “Tor Vergata”, Via Montpellier 1, 00133 Rome, Italy

**Keywords:** trans-Δ9-tetrahydrocannabinol, cannabis potency, resin, hashish, forensic toxicology, gas chromatography-flame ionization detection (GC-FID)

## Abstract

Cannabis remains the most illicitly produced and consumed substance worldwide, and the average trans-Δ9-tetrahydrocannabinol (THC) content in cannabis products (marijuana, hashish) has increased over time. This paper presents data about THC concentration in cannabis resin samples seized by law enforcement from 2015 to 2022 in the southern area of Rome (Italy). From 2015 to 2022, more than 1000 hashish samples were analyzed; the average THC content was 18.0% and dramatically increased from 13.7% (2015) to 27.1% (2022). The potency of THC in some samples characterized by unusual shape and color was higher than 24% and, in a few cases, higher than 40%. The age group most involved in seizures of cannabis resin concerned males aged between 15 and 36 years old. The spread of this phenomenon increases the risk of adverse health outcomes. Many observational studies compare the increased cannabis potency with the onset of psychosis, depression, anxiety and cannabis use disorders (CUDs), mainly in young adults. THC-potency monitoring provides data that can be helpful to create a network of communication and interaction between universities, and legislative and public health institutions to support education, awareness and surveillance related to cannabis abuse.

## 1. Introduction

Cannabis plants belong to the Cannabaceae family, and *Cannabis sativa* L., the most widespread of the species, has been used for millennia as a source of fibers, oil, food and medicine [1]. Over the years, many phytocannabinoids have been isolated from *Cannabis sativa* L. [2], and among them, the main psychoactive component of the plant is trans-Δ9-tetrahydrocannabinol (THC), which was first isolated from hashish in 1964 by Gaoni and Mechloulam [3]. In addition to THC, other major cannabinoids produced in the glandular trichomes of the plant are cannabidiol (CBD), cannabinol (CBN), tetrahydrocannabivarin (THCV), cannabigerol (CBG) and cannabichromene (CBC) [4]. In fresh plant material, these compounds primarily occur as carboxylic acids (e.g., tetrahydrocannabinolic acid A and cannabidiolic acid) endowed with pharmacological properties and no psychotropic effects [5]. Tetrahydrocannabinolic acid A (THCA-A) is a thermolabile molecule that can be converted to its psychotropically active compound (THC) when heated at high temperatures (85–100 °C) [5].

*Cannabis sativa* L., besides being a natural source of cannabinoids, is also commonly used to illegally produce herbal cannabis (marijuana) and cannabis resin (hashish, the psychomimetically active resin of the female flowering tops of *Cannabis sativa* L.) [6].

Several techniques are employed to produce cannabis resin. Hashish can be obtained by purifying the volatile oil-free alcoholic extract; with the secretion of the stalked glandular trichomes [7], which are primarily concentrated in unfertilized female flowers prior to senescence [8,9]; and by sieving the plants to reduce the material into a powder that is subsequently turned into a pressed paste-like substance coming in all shapes and sizes [10]. For the large-scale production of cannabis resin, dried plants are sieved manually or with automated equipment to improve production efficiency.

New-generation extraction methodologies involve the use of sonication, threshing with vibrating and rotating sieves, water separation, ultrasonic sieves and high-efficiency mechanical strainers [11].

Natural cannabis products are usually inhaled or taken orally [12]. Although the leaves and flowers of *Cannabis sativa* plants are usually smoked in many different ways, including cigarettes, cigars, pipes, water pipes or “blunts” (marijuana rolled in tobacco-leaf wrapper from a cigar) [13], other routes of administration are becoming increasingly common, such as vaporization, dabbing [14] (inhalation of cannabis-based concentrates and extracts) and oral consumption (cookies, candies, lollipops, cakes).

The pharmacokinetics of THC change depending on its route of administration. THC is a partial agonist at the CB1 and CB2 receptors in the endogenous cannabinoid system [15]. The concentration of CB1 receptors is predominant in the brain, whereas CB2 receptors are mainly located in the immune system.

Cannabis inhaling leads to rapid onset of psychotropic effects within seconds or minutes, reaching the peak plasma THC concentration after 15–30 min and decreasing within 2–3 h after intake. The desirable effects consist in intensified well-being and relaxation with a heightening of everyday sensory experiences; on the other hand, the most relevant acute adverse effects caused by overdosing are anxiety and panic attacks, and concerning somatic effects such as heightened heart rate and changes in blood pressure [16].

The differences in THC metabolism and psychotropic effects are mostly attributed to intra- and inter-subject variability in inhalational characteristics; the highest THC concentration turned out to be greater in frequent smokers than occasional smokers, most likely due to a more efficient smoking technique by frequent smokers [16].

Although it is widely used both for its relaxing or euphoric effects, as well as increased perception of external stimulation and its sedative, anxiolytic action, it might cause thought disorders [17], cognitive distortions, difficulty in concentration, psychomotor retardation, impaired judgment and attention, and acute impairments of both cognitive and psychomotor performance [18]. Cannabis use may also lead to the development of withdrawal symptoms and addiction in about one-tenth of users [18,19].

Despite the serious side-effects following heavy and prolonged consumption of cannabis-based products and the risks associated with long-term cannabis exposure, such as cardiovascular disease, stroke [20,21] and cannabinoid hyperemesis syndrome (CHS) [22], cannabis use does not appear to be the main or direct cause of death in fatal cardiovascular events only apparently linked to cannabis consumption [23].

### New Changes in Cannabis Potency

Cannabis remains the most illicitly produced and consumed substance worldwide in relation to the geographic areas of cultivation, the amount of material produced and seized, and the age groups of users cross-distributed within the global population [24,25].

The potency of cannabis has increased dramatically over recent years, resulting in negative impacts in term of health outcomes and psychological effects [26].

The United Nations Office on Drugs and Crime (UNODC) estimates that the overall number of the global population who used cannabis in recent years has increased by nearly 18% over the past ten years (2010–2019) [27]. The European Monitoring Centre for Drugs and Drug Addiction (EMCDDA) in 2022 also registered an increase in cannabis use in the European Union (EU) across all age groups, especially among young adults aged 15 to 34 years, with an incidence rate of 15.1% [28].

In 2019, 7.8% of the adults aged 15–64 years (25 million people) consumed cannabis in the previous year, and most of the users (about 60.0%) were males under 35 years [26].

According to the European Drug Report published in 2021 by the EMCDDA, the number of cannabis users in Western and Central Europe rose up to 6.0% to 8.0% over the previous decade (2010–2019), and an increase in the use among adolescents has been widely observed [29].

Over the last years in the EU, the average THC concentrations (potency) in resinous material significantly increased (12–29%), differently from findings on marijuana preparation (7–13%) [25,28].

The aim of this paper is to report and examine data about THC concentration of cannabis resin samples seized by law enforcement and analyzed by the Forensic Toxicology Laboratory of “Tor Vergata” University of Rome from 2015 to 2022.

The data presented in our study clearly highlight the widespread use of cannabis resin products characterized by high potency, and distinctive shape, appearance and color in Italy. Due to the large sample size analyzed, the long observation period and the results collected, our study can be useful in informing the authorities, health professionals and the scientific community about the change and spread of new narcotic substances with high THC potency.

## 2. Materials and Methods

**Figure 1:**
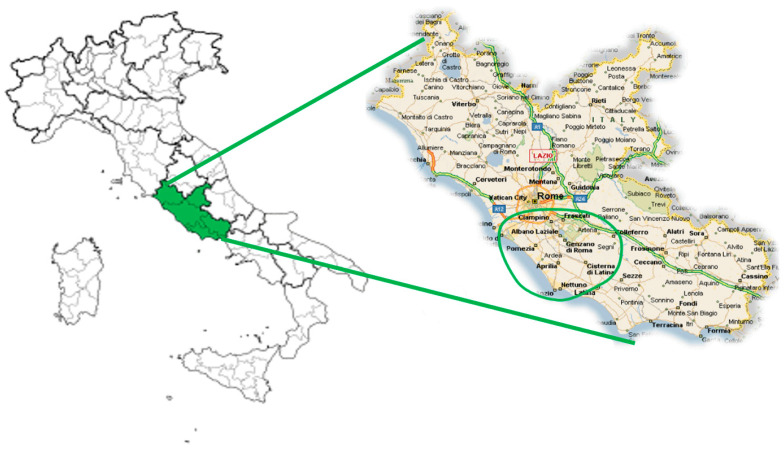
The green circle circumscribes the geographical area investigated in the study **(Lazio Region, Italy)**.

The resin materials were seized by police forces in the southern metropolitan area of Rome (Italy) as a result of the violation of Article 73, Presidential Decree No. 309/90 (Italian Law governing criminal offense for the detention, use and dealing of illicit substances). The competent judicial authority covers a total population of over 650,000 residents, divided into 30 municipalities (Figure 1).

A total of 1002 samples of cannabis resin, seized in the described area, were analyzed in our laboratory between June 2015 and December 2022.

In our laboratory, qualitative–quantitative analyses were carried out to identify the presence of illicit substances and to determine whether those substances were scheduled according to the Italian regulation on narcotics and drugs (Presidential Decree No. 309/90 and subsequent amendments).

These analyses, as requested by the judicial authority, must provide information about the percentage by weight of the active ingredient, number of doses and the average daily dose (DMG) that can be obtained from the seized material [30]. The data were collected from chemical–toxicological consultancies sent to the judicial authority as part of criminal proceedings and from analytical reports kept in our laboratory’s electronic archive.

The documentation only relates to criminal prosecutions that did not involve people who possessed small amounts of illicit substances.

Microsoft Excel (Microsoft Office Professional Plus 2016) and GraphPad by Dotmatics (GraphPad Software, Prism 9.1.1 Software, San Diego, CA, USA) were used to process all the data.

### 2.1. Standard Solutions and Reagents

Methanol (ACS reagent, reag. ISO, reag. Ph. Eur., ≥99.8% for GC) and dibucaine hydrochloride (analytical standard, ≥99%) were obtained from Merck KGaA (Darmstadt, Germany). Certified reference material (CRM) of THC solutions (10 mg/mL in ethanol) was purchased from Lipomed AG (Fabrikmattenweg 4, CH-4144 Arlesheim, Switzerland).

### 2.2. Sample Description, Preparation and Analysis

**Figure 2:**
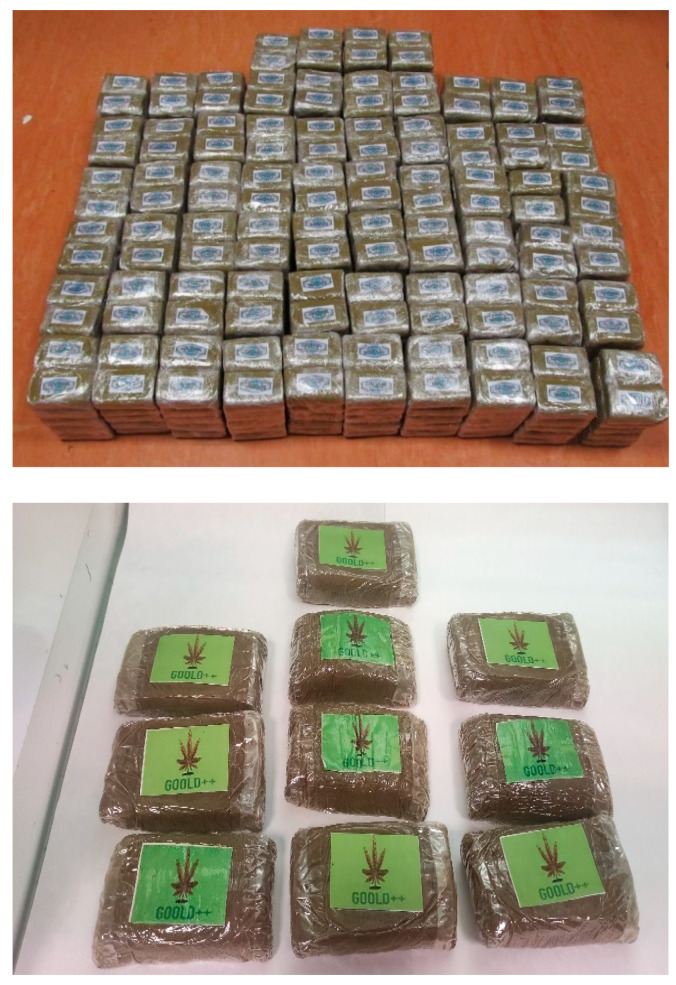
Hashish blocks wrapped in transparent film and labeled with catchy names and pictures.

The resinous material was mostly in the form of slabs or blocks, concealed in cases or reduced to small pieces and sold in small transparent plastic bags (Figure 2).

Samples were initially arranged according to their morphological and packaging characters. The net weight of each item was determined after removing the packaging, and an aliquot of 100 mg was taken from each sample. The aliquots thus obtained were then mixed thoroughly to obtain a representative sample of the total seized (pool).

From the pool, a variable number of samples, statistically significant in relation to the size of the seizure, with weights ranging from 40 mg to 60 mg, were analyzed [31].

Qualitative and quantitative analyses were performed using gas chromatography with flame ionization detection (GC-FID) validated method to assess the percentage of THC content, after dissolving the resinous material in 4 mL of methanol containing dibucaine 2 mg/mL as internal standard (ISTD).

The dissolved samples were briefly vortexed and sonicated for 20 min at room temperature. The samples were centrifuged at 4500× *g* for 10 min and filtered through a 0.2 µm PTFE syringe filter (Thermo Scientific^TM^, Waltham, MA, USA). Then, 1 μL of each sample was injected into the GC-FID system (OpenLAB CDS Chemstation Edition, Agilent Technologies^©^, Palo Alto, CA, USA. Software Driver Version 5.05 Rev. C.01.07 [27], MSS Driver Version 1.0.3.0, 2001–2014).

The GC-FID instrumentation consisted of an Agilent 7820A GC system and an Agilent G4513A series autosampler (Agilent Technologies, Palo Alto, CA, USA). The column was an Agilent HP-5 fused silica capillary column of 30 m in length, 0.32 mm in ID, and 0.25 μm in film thickness (Agilent Technologies, Palo Alto, CA, USA). The carrier-gas (nitrogen; N2) flow was constant at 1 mL/min.

**Figure 3:**
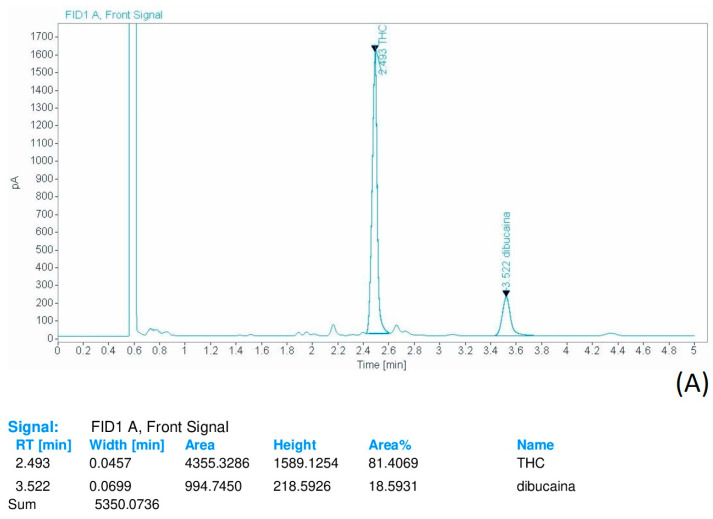

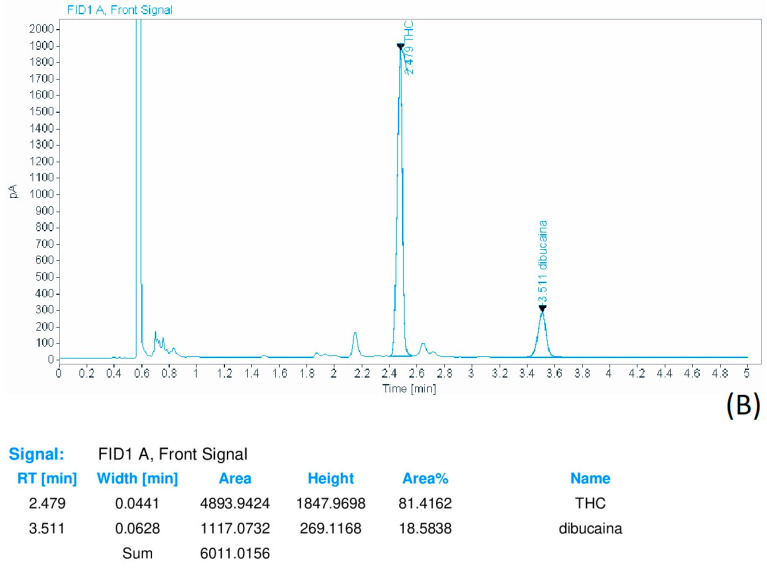
(**A**,**B**) Representative chromatograms of GC-FID analysis of THC resin samples.

Each sample was injected into the gas chromatography system using a 5:1 split injection ratio. The total run time was 5 min (Figure 3A,B).

The oven temperature was set as follows: the starting temperature of 200 °C was held for 0.5 min, then increased to 260 °C at a rate of 15 °C/min and held for 4 min. The injector temperature was 290 °C.

At this temperature, the decarboxylation of THCA-A to THC can occur, contributing to the total THC amount [32,33].

The decarboxylation process may depend on various factors, such as thermal and desiccation conditions, exposure to sunlight, lipophilic environment, length of processing and solvents used for the extraction procedure [34,35,36].

The contribution of THCA-A was not separately calculated. This represents an obvious limitation of our study caused by an initial low availability of the certified standard of THCA-A. The thermal conversion of THCA-A to THC represents a crucial aspect that requires further investigation. For this reason, the development of a method for the isolation, determination and quantification of THCA-A is already planned.

## 3. Data Elaboration and Results

The number of hashish samples for each year was relatively stable from 2016 to 2022 ($\bar{x}$ = 18.7).

For each year, mean, median, minimum and maximum THC concentrations were calculated and are summarized in Table 1.

The data reported in Table 1 show that the number of samples seized and analyzed was constant throughout the study period, with a minimum number of samples in 2015 (*n* = 67) and a maximum number of samples recorded in 2020 (*n* = 145), despite restrictions due to the COVID-19 pandemic.

**Figure 4:**
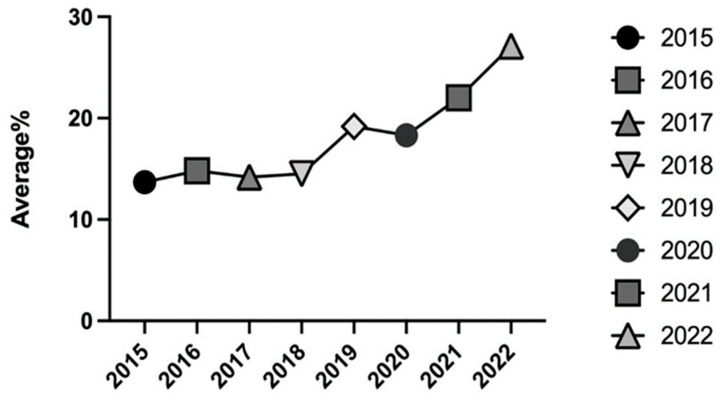

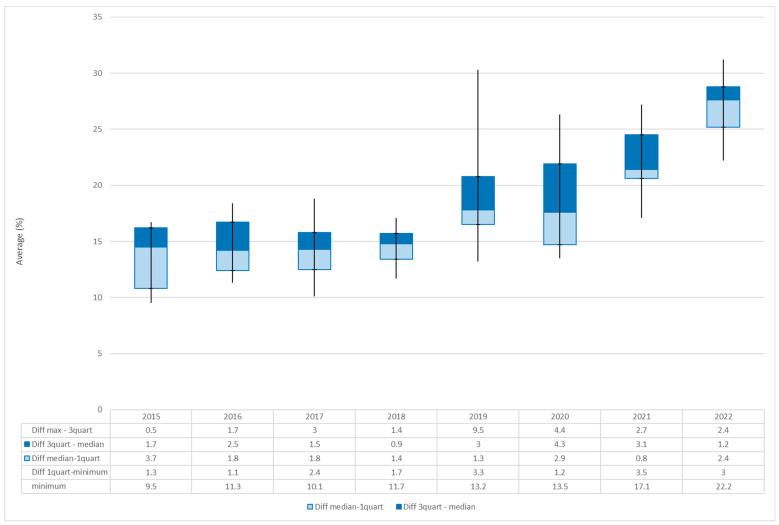
Average percentages of THC content (%) in samples seized between 2015 and 2022.

The mean THC concentration measured over the 8-year study period reported in Figure 4 shows an increasing trend across all the study period, ranging from 13.7% in 2015 as a minimum value to a maximum value of 27.1% in 2022.

In 2019 and 2020, a first increase in THC concentration was observed, with mean values of 19.0% in 2019 and 18.0% in 2020; then, further increases were recorded in 2021, with a mean value of 22.0%, and in 2022, when a peak average concentration value of 27.0% was registered (Figure 4).

Data analysis focused on samples with a high THC concentration. The analytical results were compared with data reported by EMCDDA in 2021 and in the “Parliament 2021 Report on drug addiction in Italy”. In these documents, the percentage values of THC potency between 20 and 28% (calculated mean value of 24%), and between 30 and 50% (calculated mean value of 40%), respectively, were recorded. Consequently, we split our data into two separate groups, and the subsets of samples were analyzed separately; the first subgroup included samples with a THC percentage ≥ 24.0% (Group I), whereas the second subgroup included those with a percentage ≥ 40.0% (Group II) [37].

Group I consisted of 75 samples of hashish seized mostly from 2019 to 2022. The minimum THC percentage was recorded in 2020 (24.3%), whereas the maximum value was recorded in 2019 (44.1%).

**Figure 5:**
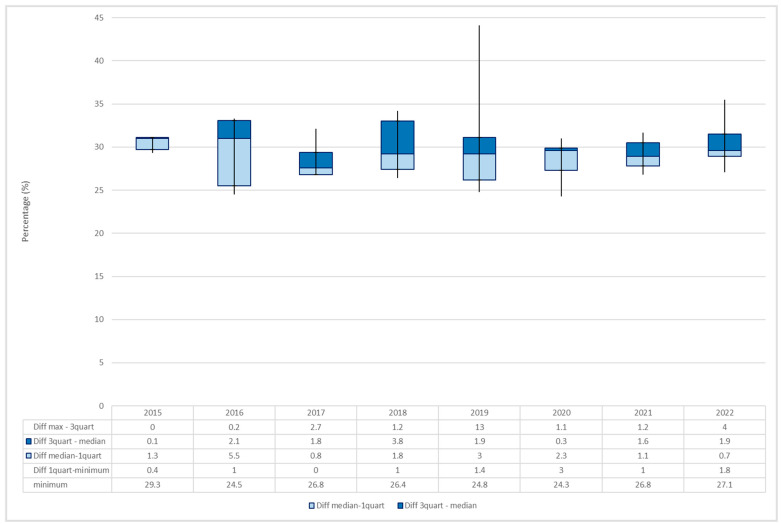
Average percentages of THC content (%) in seizure samples with a THC percentage ≥ 24%.

By examining the average percentage for each year, a variation over the years was observed, with the maximum value being registered in 2015 (30.6%). The increasing trend was interrupted by a slight decrease in the following three years, followed by additional increases in 2019 (30.1%) and in 2022 (30.2%) (Figure 5).

Group II consisted of 15 samples mainly analyzed from 2019 to 2022, characterized by an average THC percentage of 48.6%, with a minimum of 40.2% (December 2020) and a maximum of 66.6% (July 2022). The number of samples with the highest THC potency occurred in 2022 (*n* = 7), whereas during the period 2016–2018, no samples with a THC percentage > 40% were recorded.

The seized samples with the highest THC content had particular appearance, shape and color compared with those most frequently observed, which were mostly dark-brown, solid-shaped blocks or slabs.

**Figure 6:**
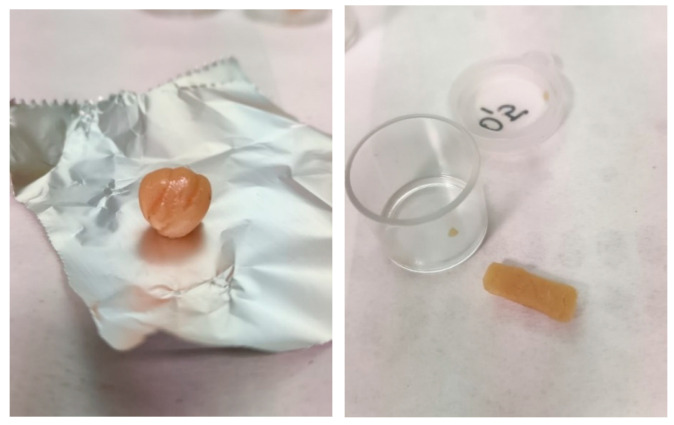

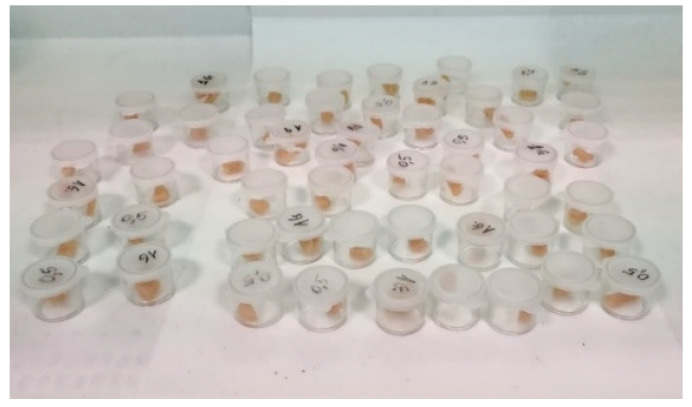
An amber, round-shaped hashish sample with a high THC concentration (51.2%).

In particular, in October 2021, we analyzed samples of cannabis resin with a rounded or rectangular shape of light amber color that had an average THC content of 51.2%. (Figure 6).

**Figure 7:**
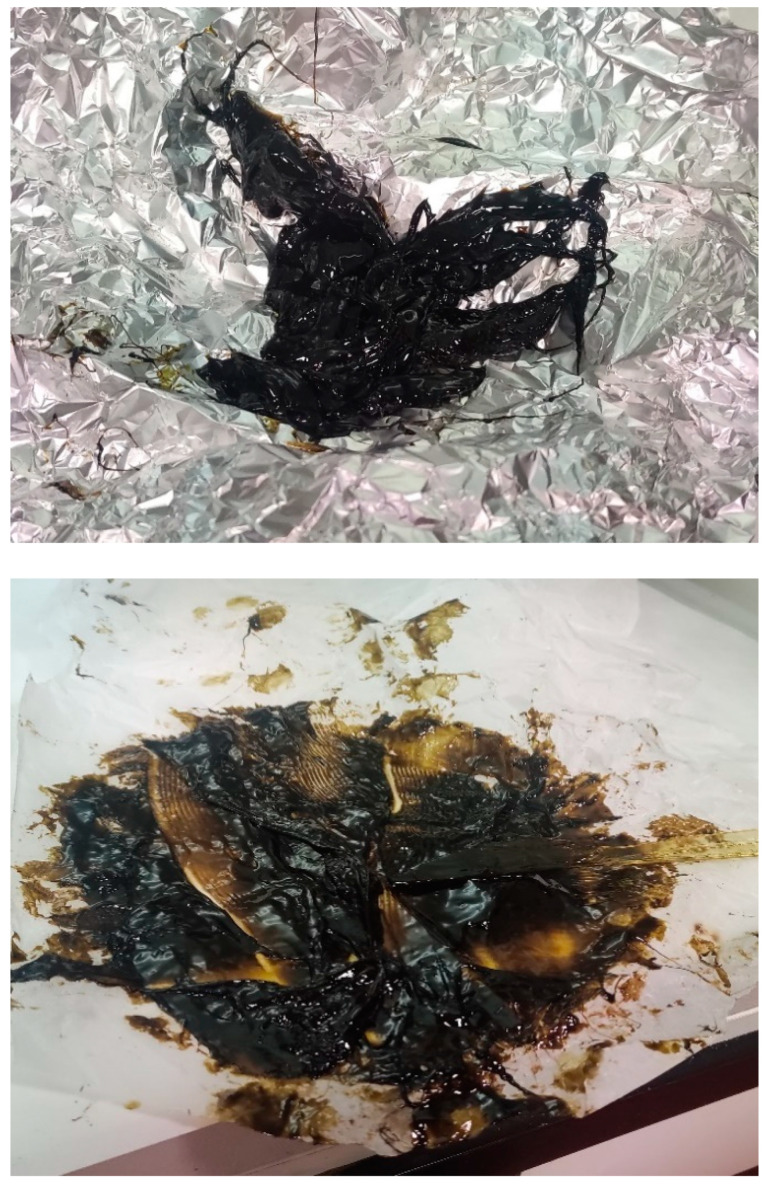
A sticky hashish sample with a THC concentration greater than 60%.

Another case of highly concentrated hashish occurred in July 2022 when three wrappers containing a dark-brown resinous substance with a soft, sticky consistency similar to a spreadable substance with an average THC percentage of 66.6% were seized (Figure 7).

### Epidemiologic Trends in Cannabis Resin-Related Arrests

The individuals arrested during the study period due to the violation of Article 73, Presidential Decree No. 309/90, for cannabis resin possess were 1238.

Most seizures involved males (*n* = 1127, 91.0%), whereas females were a small minority (*n* = 111, 9.0%). Adult consumers (aged 18–79) were on average 32 years of age for males and 35 years of age for females. The lowest age observed in the female group was 19 years old. The males were the youngest, with average ages of 17 years (2015–2016) and 16 years (2017), and the youngest stopped by law enforcement was 15 years old (2019).

Most of the seizures involved people younger than 36 years. The 26–36 age group included most of the individuals arrested (*n* = 383, 34.6%), whereas a percentage of 32.3% (*n* = 358) was included in the 15–25 age group (Table 2).

## 4. Discussion

The purpose of our study was to examine THC content in seized samples to better understand the phenomenon of the increasing THC potency in cannabis resin.

The samples seized in breach of Article 73 of Presidential Decree No. 309/90 can provide important information on the purity of the drug purchased on the black market.

The analyses carried out on 1002 hashish samples seized by police forces and sent to our Forensic Toxicology Laboratory between 2015 and 2022 highlighted an average potency value of THC content in cannabis resin of 18.0%.

The average potency of cannabis resin from 2015 to 2019 was stable, whereas from 2019 to 2022, the data show an interesting increase in its potency (+29.2%).

Data obtained from our study and in particular, those regarding the highest percentages of THC were forwarded to the National Early Warning System (S.N.A.P.) [38] in order to issue an alert on new forms of production, packaging and sale of products with high THC content.

Our data are in agreement with national and European ones [37,39].

The “Parliament 2022 Report on drug addiction in Italy” reported that cannabis production is increasing, and cannabinoid formulations have been changing in recent years. Recent data from Italian police forces highlight a constantly increasing trend in mean THC content in seized samples of cannabis resin (25–26%, up to 30%) (Table 3).

The THC values were below 10.0% until 2016. In 2019, the average and median THC contents reached a percentage of 20%, and in the last two years (2020–2021), data reported a further increase in THC content up to 25% [37].

Data reported by the Department for Drug Policies (DPA) also revealed a steady increase in potency of seized cannabis products. The average percentage of THC detected in hashish samples is almost double (24–25%) as that of THC detected in marijuana samples (10–12%). Recently, there have been seizures of high-THC products (from 35% to 50%), and we also highlighted an increase in the number of seizures of high-THC content samples of over 70% [39,40].

European data on cannabis potency collected by the EMCDDA from the 28 EU member states, Norway and Turkey also show the increment in cannabis resin potency, with values ranging from 20% to 28% [29].

Furthermore, cannabis use is significantly higher in the 15–24 age group, as reported by the EMCDDA in a study including 26 countries between 2015 and 2020 [29].

These data match those reported in our study and other data provided by the DPA in 2022 in “Report on drug addiction in Italy” [39].

In our study, the age group most involved in the seizures of cannabis resin concerned males aged between 15 and 36 years old.

According to the Annual Report on Drug Addiction in Italy, in 2021, cannabis use was more prevalent among young adults aged 18–29 [28]. In particular, 16.0% was included in the age group 25–29, 22.6% in the age group 20–24 and 5.9% was under the age of 18 [39]. Moreover, cannabis was also the most used substance in Italy in 2022 among students; in particular, 85.6% was in the form of weed/marijuana, and 51.0%, resin products [39].

The reported data reveal the association between the dramatic increase in cannabis potency and the decrease in the age of people arrested for dealing and/or possession of cannabis.

The spread of this phenomenon increases the risk of adverse health outcomes. Many observational studies compare the increased cannabis potency to the onset of psychosis, depression, anxiety and cannabis use disorders (CUDs), mainly in young adults [41,42,43]. Therefore, it is critical to implement strategies to prevent the rise in these illegal substances to protect public health and raise awareness of the risks of their use.

## 5. Conclusions

This study examined patterns of increase in THC potency of cannabis resin seized in an eight-year study period (2015 to 2022).

Despite the restrictive measures and stress combined with social isolation caused by the COVID-19 pandemic, the production and sale of illicit substances has not been stopped.

The cultivation of cannabis and the production of synthetic drugs in the EU continued throughout 2020 despite all the restrictions imposed on the world population [29], leading to an increase in the cultivation of cannabis plants at home [44].

Significantly, our Forensic Toxicology Laboratory, in 2020, pointed out an increase in cannabis resin seizures by Italian law enforcement agencies.

Although this study only involves an area of the Italian territory, our data can be overlaid with European and global ones.

Starting from this analysis, we identified a progressive and significant increase in THC potency of cannabis resin. Thus, following the reasoning set forth by our data, we show conformity with national and international trends.

In conclusion, using data collected between 2015 and 2022 on 1002 seizures of cannabis products, we show higher potency of cannabis resin and its increasing prevalence. This phenomenon is clearly associated with severe acute and medium-term risks to human health.

THC-potency monitoring provides data that can be helpful to create a network of communication and interaction between scientific facilities involved in forensic analysis, and legislative and public health institutions to support education, awareness and surveillance related to cannabis abuse.

## Figures and Tables

**Table 1:** Number of analyzed samples (*n*), and minimum (%), maximum (%) and mean THC (%) potency of samples seized between 2015 and 2022 in the southern area of Rome.

	*2015*	*2016*	*2017*	*2018*	*2019*	*2020*	*2021*	*2022*
*Sample size*	67	141	127	138	122	145	138	124
*Minimum*	9.5	11.3	10.1	11.7	13.2	13.5	17.1	22.2
*25% percentile*	10.8	12.4	12.5	13.4	16.5	14.7	20.6	25.2
*Median*	14.5	14.2	14.3	14.8	17.8	17.6	21.4	27.6
*75% percentile*	16.2	16.7	15.8	15.7	20.8	21.9	24.5	28.8
*Maximum*	16.7	18.4	18.8	17.1	30.3	26.3	27.2	31.2
*Range*	7.2	7.1	8.7	5.4	17.1	12.8	10.1	9.0
*Mean*	13.7	14.6	14.2	14.5	19.2	18.3	22.0	27.1
*SD*	2.7	2.5	2.2	1.7	4.9	4.0	2.9	2.5
*Std. error of mean*	1.0	0.7	0.6	0.5	1.4	1.2	0.8	0.7

**Table 2:** Summary of minimum, maximum and mean ages of all the individuals arrested for cannabis resin holding between 2015 and 2022.

		Males (%)	Females (%)	Sample Size (*n*)
**2015**	Total	82 (91.1%)	8 (8.9%)	90
	Mean Age	33	32	
	Minimum	17	19	
	Maximum	65	41	
**2016**	Total	177 (92.2%)	15 (7.8%)	192
	Mean Age	32	38	
	Minimum	17	19	
	Maximum	67	72	
**2017**	Total	143 (91.1%)	14 (8.9%)	157
	Mean Age	32	39	
	Minimum	16	19	
	Maximum	60	58	
**2018**	Total	151 (89.3%)	18 (10.7%)	169
	Mean Age	32	40	
	Minimum	18	20	
	Maximum	79	59	
**2019**	Total	137 (91.9%)	12 (8.1%)	149
	Mean Age	32	35	
	Minimum	15	19	
	Maximum	70	59	
**2020**	Total	168 (91.8%)	15 (8.2%)	183
	Mean Age	33	35	
	Minimum	18	21	
	Maximum	72	66	
**2021**	Total	146 (86.9%)	22 (13.1%)	168
	Mean Age	32	35	
	Minimum	18	19	
	Maximum	64	67	
**2022**	Total	123 (94.6%)	7 (5.4%)	130
	Mean Age	32	25	
	Minimum	19	25	
	Maximum	57	27	

**Table 3:** Number of seized samples and THC content (%) in cannabis resin between 2015 and 2019 as reported in “Parliament 2022 Report on drug addiction in Italy”.

** No. of Samples **	2015	2016	2017	2018	2019	2020	2021
	1030	1505	2128	2255	2534	2427	2830
	THC Content (%)						
**Minimum**	1.0	0.3	0.8	0.7	0.5	0.6	0.6
**Maximum**	35.0	39.0	55.0	67.0	62.0	78.0	74.0
**Mean**	9.7	7.4	16.0	17.0	20.0	25.0	25.0
Median	9.2	25.0	14.0	16.0	19.0	26.0	26.0

## Data Availability

The data presented in this study were obtained from the included studies and are openly available.
